# Real word challenges in integrating electronic medical record and administrative health data for regional quality improvement in diabetes: a retrospective cross-sectional analysis

**DOI:** 10.1186/s12913-022-08882-7

**Published:** 2023-01-02

**Authors:** Rukia Swaleh, Taylor McGuckin, Denise Campbell-Scherer, Brock Setchell, Peter Senior, Roseanne O. Yeung

**Affiliations:** 1 Division of Endocrinology & Metabolism, Faculty of Medicine and Dentistry, University of Alberta, Edmonton, AB Canada; 2 Office of Lifelong Learning & the Physician Learning Program, Faculty of Medicine and Dentistry, University of Alberta, AB Edmonton, Canada; 3 Department of Family Medicine, Faculty of Medicine and Dentistry, University of Alberta, Edmonton, AB Canada; 4 Alberta Diabetes Institute, University of Alberta, Edmonton, AB Canada

**Keywords:** Regional quality improvement, Administrative health data, Electronic medical record, Diabetes, Learning health system

## Abstract

**Background:**

Linked electronic medical records and administrative data have the potential to support a learning health system and data-driven quality improvement. However, data completeness and accuracy must first be assessed before their application. We evaluated the processes, feasibility, and limitations of linking electronic medical records and administrative data for the purpose of quality improvement within five specialist diabetes clinics in Edmonton, Alberta, a province known for its robust health data infrastructure.

**Methods:**

We conducted a retrospective cross-sectional analysis using electronic medical record and administrative data for individuals ≥ 18 years attending the clinics between March 2017 and December 2018. Descriptive statistics were produced for demographics, service use, diabetes type, and standard diabetes benchmarks. The systematic and iterative process of obtaining results is described.

**Results:**

The process of integrating electronic medical record with administrative data for quality improvement was found to be non-linear and iterative and involved four phases: project planning, information generating, limitations analysis, and action. After limitations analysis, questions were grouped into those that were answerable with confidence, answerable with limitations, and not answerable with available data. Factors contributing to data limitations included inaccurate data entry, coding, collation, migration and synthesis, changes in laboratory reporting, and information not captured in existing databases.

**Conclusion:**

Electronic medical records and administrative databases can be powerful tools to establish clinical practice patterns, inform data-driven quality improvement at a regional level, and support a learning health system. However, there are substantial data limitations that must be addressed before these sources can be reliably leveraged.

**Supplementary Information:**

The online version contains supplementary material available at 10.1186/s12913-022-08882-7.

## Background

The prevalence of diabetes in Alberta is increasing at a faster rate than in all other Canadian provinces [1]. Building systems whereby clinicians engage in continuous quality improvement (QI) is increasingly important to improve the clinical outcomes of people living with diabetes. Notably, the feasibility of a learning health system (LHS) and success of data-driven QI is predicated on clinicians and healthcare staff having timely access to accurate and reliable information about practice patterns and clinic processes [2–4].

Routinely collected clinical and administrative health data, such as physician claims and information stored in electronic medical records (EMRs), are a promising source of information for creating a learning health system (LHS) [2]. Specifically, they can be leveraged to inform individual clinical decision making, evaluate healthcare system structure and delivery, and serve as the foundation for medical and health services research [5, 6]. They provide opportunity for continuous rapid learning opportunities. Although convenient, there are several challenges and potential hazards of using information from these sources to inform QI as a means to bridge the gap between research and clinical practice [6].

Data from electronic medical records and administrative databases are not “fit for purpose”, meaning that the information initially served a different function than how it would be used for QI [7, 8]. For example, information that was entered for an administrative purpose such as billing would be used to quantify the prevalence of a specific disease, assess patient outcomes, or assess adherence to a clinical guideline [8]. Before using information from these sources to develop an LHS, it is imperative to consider how the initial purpose may impact the quality, completeness, and capture of information that would be needed. The reliability and accuracy may be affected by discrepancies between software design, user needs, clinical workflows, and biases such as pay-for-performance parameters, practice workload, and EMR design [5, 9]. Additionally, clinically-relevant details that are necessary for QI and to support continuous learning are often unavailable or incomplete [10]. Therefore it is vital to assess the availability, accuracy, and quality of routinely collected EMR or administrative health data before using it as the basis of an LHS or to conduct health services research, [11].

There is significant variability when it comes to what and how information is captured, collected, and stored within and between healthcare settings [9, 12]. In Alberta, efforts have been made to harmonize medical records across the province, and it has been recognized as having one of the more robust digital health ecosystems in Canada [13]. Consequently, it might be presumed that Alberta would be an ideal place to assess whether linked EMR and administrative data sources should and could be used to build and support an LHS. There are a number of initiatives in the province using either administrative health data alone such as the Diabetes Infrastructure for Surveillance, Evaluation, and Research (DISER) [14], or primary care EMR data such as the Canadian Primary Care Sentinel Surveillance Network (CPSSCN) [15]. Additionally, linked EMR and administrative data have been used in diabetes care and health services research outside of Alberta to inform strategies for more effective chronic disease management, for instance, to create algorithms to identify more detailed subgroups [16, 17]. However, none have assessed the linking of EMR and administrative data for QI, specifically in the context of specialty diabetes care. In this study, we aimed to assess and report the process, feasibility, and limitations of linking EMR and administrative data sources in Alberta for the purpose of QI within diabetes specialty care.

## Methods

### Study setting and design

A retrospective cross-sectional analysis was conducted using data from the EMR (eClinician, Epic Systems Corporation, Wisconsin), and Alberta Health Services (AHS) administrative databases (AHS Labs, Physician Claims, Pharmaceutical Information Network (PIN)) for individuals ≥ 18 years old attending the five multidisciplinary specialist diabetes clinics across the Edmonton Zone. Each clinic is staffed by specialist physicians (internists and endocrinologists), registered nurses, and registered dietitians, with the majority of the allied staff being Certified Diabetes Educators. Data were extracted for visits made between March 2017 to December 2018. March 1, 2017 was chosen as the start date as this was a few weeks after the EMR went live at all the clinics. For laboratory data, inclusion dates were expanded to include 2014 to 2018 in order to get a more complete data set.

Ethics approval was obtained from the Health Research Ethics Board - Health Panel at the University of Alberta (Pro00085385) and all methods were performed in accordance with the relevant guidelines and regulations established by the committee. The need for written informed consent was waived by the Health Research Ethics Board - Health Panel ethics committee due to retrospective nature of the study. This research was conducted in accordance with the principles of the Declaration of Helsinki.

This work was conducted in partnership between the University of Alberta Division of Endocrinology & Metabolism, Alberta Health Services (AHS), and the Physician Learning Program (PLP). The Physician Learning Program is an Alberta Government funded program that works with partners to co-design improvement initiatives to address healthcare gaps [18]. Alberta Health Services’ Edmonton Zone Diabetes Quality Council is a regional initiative to improve diabetes care and includes members from five multidisciplinary specialist diabetes clinics and key community stakeholders including community diabetes educators representing major regional primary care networks.

### Study Aims

1. Describe the processes arising while linking and analyzing administrative and EMR data to obtain information on practice patterns and clinical outcomes within diabetes specialty clinics for the purpose of QI.
2. Perform limitations analysis to determine questions that can be answered and those that cannot be accurately answered using these linked data sources.

### Methodological process of linking EMR and administrative data 

Clinical partners working within the diabetes clinics were responsible for formulating questions of interest. Team members from PLP were responsible for extracting, integrating, and synthesizing data. The variables needed to address clinical questions were identified. This included patient demographics, service use information (e.g., duration of visit), type of diabetes, anthropometrics (e.g., body mass index), clinical and laboratory parameters (e.g., Hemoglobin A1c), and diabetes complications (e.g., diabetic retinopathy) (listed in full in Appendix 1). Data sources that housed the relevant information were identified and included the EMR used across all the specialist clinics and AHS administrative databases (Appendix 2). A data query was formulated by a trained AHS data analyst, clinicians who use the EMR, and administrative data experts. Data were extracted using case definitions for each comorbidity, laboratory codes for tests of interest, and Anatomical Therapeutic Codes (ATC) for medications. Appendix 3 describes the Alberta Health International Classification of Disease (ICD) 9 codes used to define and capture comorbidities. The datasets were linked using a common identifier, a personal health number (PHN). Oracle SQL Developer was used to match and prepare the data. Data cleaning and analysis were conducted using Python 3.4 (www.python.org). Descriptive statistics of the clinic service provision and population characteristics are presented in Appendix 4 for contextual purposes only, but are not the focus of this paper.

### Limitations analysis

While analyzing the data, clinician partners and data analysts identified potential discrepancies between the data that was extracted and what the clinicians working at the clinics expected. People with intimate knowledge of the clinical workflows (i.e., clinicians and clinic staff), AHS data analysts with extensive experience in the administrative data landscape in Alberta, and data experts with deep knowledge of administrative health data, determined that the extracted and analyzed data likely did not accurately represent and capture clinical care. For example, the proportion of patients with blood pressure readings was much lower than expected considering there is dedicated staff at the clinics who take these readings for each in-person visit. After the analysis was complete, a limitations analysis was conducted to group clinical questions into three categories: answerable with confidence, (2) answerable with limitations, (3) and non-answerable using the available data sources. For questions that were answerable with limitations, the team further investigated reasons why the data may be incomplete.

## Results

### Process of mobilizing clinical information from administrative and EMR Data to inform Quality Improvement

**Fig. 1:**
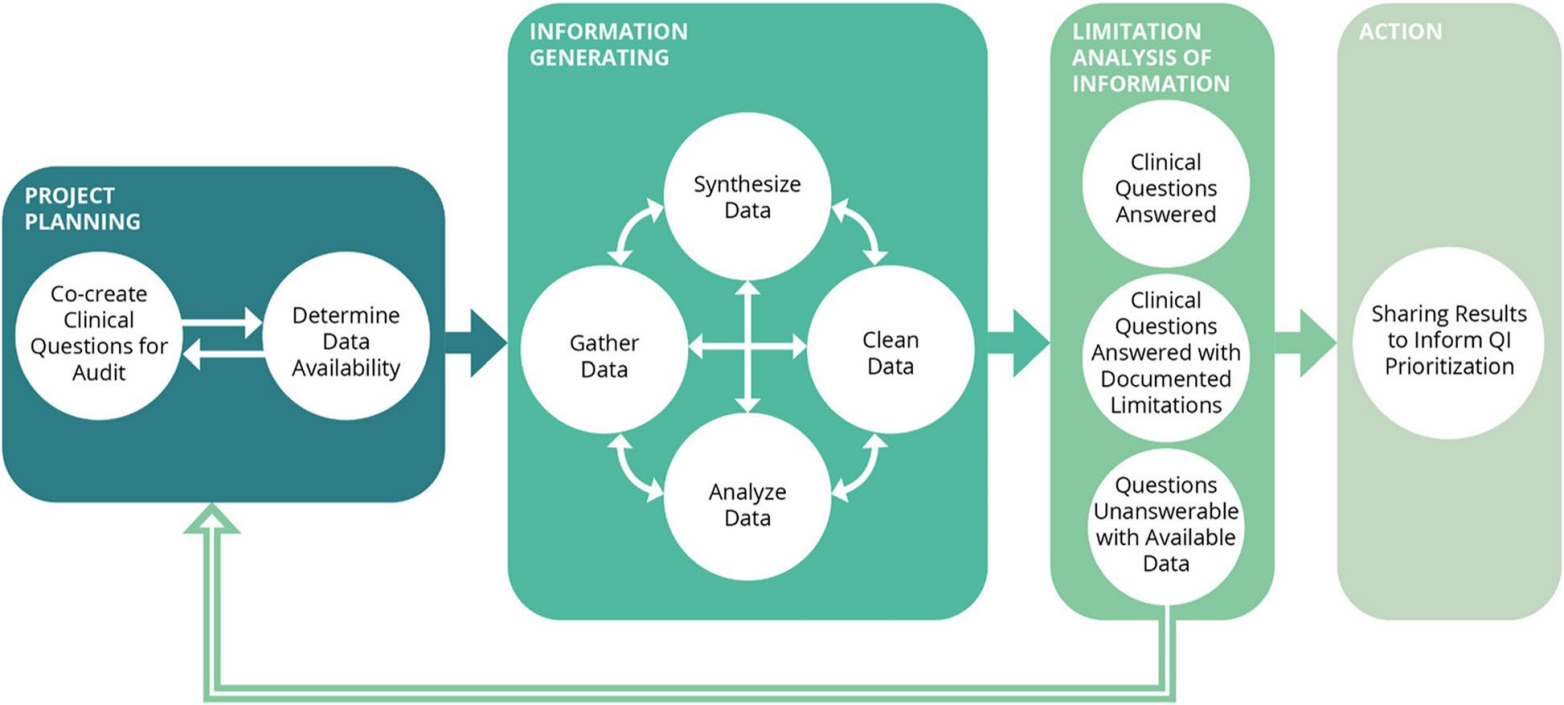
The process of mobilizing clinical information to inform quality improvement using administrative and EMR data

The process of utilizing EMR and administrative data to drive QI initiatives for diabetes care was found to be iterative rather than linear and is outlined in Fig. 1.

There were four overarching phases that the project team moved through from project conception to action. The four phases were:

1. Project planning: In this phase, clinical questions were identified by a multidisciplinary project team including clinicians from the various specialty clinics, other clinical staff, and data analysts. This was done to ensure the questions were clinically relevant and practical. After identifying clinical questions, the analysts determined data availability. This process was iterative as the information needed to answer the questions had to be available in the shared EMR and administrative databases.
2. Information generating: Once the clinical questions were identified, the data was collated. This required the collective effort and expertise of the multidisciplinary team members as the data was often unstructured and located across several databases. When the variables required to answer the clinical questions were not in a database, other existing data fields were queried and reasons as to why the variables were not available were explored. For example, to assess whether an individual had a lifetime history of cardiovascular disease, we looked to whether they had a record of visiting a healthcare facility with the relevant ICD code. Limitations and considerations during this assessment included how long the database had been in existence and whether the individual had lived in the province for the entirety of the time that the database was available. The collated data was then cleaned and synthesized. This was an essential part of the analysis process as the data was often unanalyzable in the extracted format. For example, free text was heavily used in some instances making descriptive statistics impossible to compute without thoroughly cleaning the data field. There were also cases where results were captured in a non-numeric format. For example, lab results were reported as “<2.22 mg/mmol” for albumin:creatinine ratio as opposed to an absolute value. Simply removing all non-numeric characters or assigning a null or zero value to calculate a mean or number of missing values, would have led to inaccurate analyses and/or imprecise conclusions. Programming languages were central to the data cleaning and synthesizing process.
3. Limitations analysis of information: After the data was analyzed, a limitations analysis was conducted and clinical questions were grouped into three categories: (1) could be answered with no concerns, (2) could be answered but with concerns that limit the usability of the data, and (3) could not be answered with the available data and resources. Table 1 outlines the types of questions classified in each category and is presented in more detail below.
4. Sharing results to inform quality improvement prioritization: The results from the first three phases were presented to the Edmonton Zone Diabetes Quality Council. It became clear that part of the QI priorities included improving data capture so that more clinical questions can be answered with confidence and can be monitored over time to assess clinic practice patterns and patient outcomes. The Council is working to develop processes to act on the presented data.

**Table 1:** Limitations, validity, and quality analysis of the data to categorize the answerability of questions

	Sample questions	Description of Concern (if applicable)
**Could be answered with confidence**	Number of unique individuals and total outpatient visits	n/a
	Age	n/a
	Sex	n/a
	Types of visits (in person vs. virtual)	n/a
	Laboratory markers	n/a
**Answered but with documented limitations**	Proportion of individuals with at least one lipid panel result recorded	Proportion of individuals with non-HDL levels recorded was lower compared to other lipid profile components, even when parts of the lipid panel are drawn and reported as a unit and not standalone tests. After consulting with the clinical biochemist, it was determined that the discrepancy is likely due to lab reporting practices. Prior to 2016, non-HDL levels were not provided by the lab and required the clinician to calculate the result manually. After 2016, there was a change in practice and the lab now reports non-HDL levels automatically. As laboratory data were analyzed between 2014–2018, non-HDL levels would not have been captured in the former half of the results, hence explaining the lower than expected proportion.
	Diabetes type	Almost 1 in 4 individuals had multiple conflicting types of diabetes recorded in the EMR. These individuals are coded as “uncertain diabetes type” in our analyses as it is impossible to determine the true diagnosis without an in-depth chart review. The accuracy of coding was questioned as Diabetes in Pregnancy and Gestational Diabetes Mellitus (GDM) were both coded in males (n = 12). It is possible that some of the males documented to have GDM may have been transgender individuals.
	Proportion of in-person visits with blood pressure measured	Lower than expected at a clinic where there are nurses dedicated to recording blood pressure for every in-person physician visit. This may possibly be a data entry or data capture error; we could not carry out effective data mapping to explain the discrepancy due to resource constraints.
	Proportion of in-person visits with height and weight measured	Lower than expected as the dedicated nurses also measure height and weight. BMI measurements may have been missing as not all individuals had a height recorded within the study period. This may be due to height only being checked at the initial visit and not carrying forward past 365 days from previous encounters in the database to enable BMI calculation in subsequent encounters.
	Length of appointments	Appointment lengths were available for clinic visits, appointments, consult letters, and chronic disease management. These were based on how long the appointments were booked for and not how long the practitioner actually spent with the individual. Some visit types, such as half-day (or longer) classes, will inflate the mean appointment lengths.
	Comorbidities and complications	Identifying comorbidities and complications in both the EMR and administrative data proved to be difficult. Literature was consulted to find case definitions used by other researchers, clinicians, and organizations (e.g., NAPCReN [19]) whenever possible. However, we also used a single instance of a certain ICD code across a number of databases to identify the presence of a comorbidity or history of a complication. Uncertainty of the comorbidity data in the EMR arose because information can be recorded in the problem list or encounter table and not always both. We acknowledge that comorbidities and complications may be over- or under captured based on the definitions used.
**Could not be answered**	Duration of diabetes	Data regarding the duration of diabetes could not be extracted as there was no reliable data field available to answer this question. Accuracy is questionable as the EMR defaults to record the date when entry of diagnosis was put into the EMR if no date is specified. Historical data from the previous EMR was not merged into the new EMR when it was adopted by all clinics in 2017. As well, this variable is primarily completed via patient-physician conversations and is often not documented in the extractable data field. Finally, external data sources do not feed into the EMR to populate this field.
	New versus follow-up visits	No consistent indication/coding in EMR.
	Proportion of individuals with hyperglycemic hyperosmolar state (HHS)	HHS diagnosis is not captured within the Alberta Health ICD coding scheme.

### Limitations analysis

There were a number of concerns in the interpretation of the data as outlined in Table 1. After completing data analysis and reviewing results with content experts, we grouped clinical questions into those that could be answered with confidence, those that could be answered but accuracy and usability of data were questioned, and those that could not be answered at all. Questions that were answerable with confidence were those where data was available in either administrative databases or EHRs and the data was determined to be reliable based on the reported numbers. Questions that were answerable with limitations included those where we captured some information, but there were concerns about the completeness and/or accuracy. This led us to question whether the results could be reliably used for QI. Other questions were classified as non-answerable if there was no information available in any of the queried databases.

## Discussion

Developing an LHS has the potential to improve patient outcomes by supporting clinical decision making, assessing adherence to clinical pathways, and providing opportunity for continuous improvement. Routinely collected healthcare data has the potential to serve as the foundation in which an LHS can be built. We assessed the feasibility of integrating clinical information from EMR and administrative databases to build a regional diabetes QI strategy. In doing this, we defined the iterative process of doing this type of work.

The process of obtaining, integrating, and analyzing data from various clinical information systems was more challenging and resource-intensive than expected. Our findings raised questions about the quality and reliability of data stored in these systems. To use these information sources to create an LHS requires drastic improvements in data quality. The limitations we uncovered stem from several sources such as data fields being used inconsistently within the EMR, data not carrying forward beyond 12 months due to EMR design limitations, inaccurate or inconsistent coding practices by the user, and changes in laboratory reporting practices over time. We found that some important clinical questions could be answered with confidence using the available data where others could be but with limitations. Finally, there were clinical questions that could not be answered as the information required to answer the question was not routinely collected. Understanding that status of available versus unavailable and reliable versus unreliable data is a first step to identify opportunities for QI within the diabetes specialty clinics. Overall, we found that linking EMR and administrative data to understand practice patterns and clinical processes to support an LHS for data-driven QI at a regional level was possible with dedicated resources, but that it may be limited in a routine clinical setting.

The challenges we identified in linking EMR and administrative data for QI have been documented in prior studies. We encountered limitations at every stage, from data acquisition, formatting, integration, and cleaning, to interpretation [20]. Lack of data standardization, missing or incomplete data, and incompatible representation of data were prominent issues and are not unique to our context [12, 21, 22]. We also encountered a lack of available staff and resources to enable robust data mapping, EMR design barriers, and competing priority areas for improvements in data quality within and across specialties which have been described elsewhere [23, 24]. As the data was collected for a different purpose than QI, clinical information was often unavailable, incomplete, or potentially unreliable [5]. Notably, these real-world challenges were apparent in our region, despite the relatively robust digital health ecosystem in Alberta [13]. A recent paper by our group describes some of these challenges in more detail, across other clinical contexts in addition to specialist diabetes clinics [25].

With the availability and increasing adoption of clinical information systems in healthcare settings, it is prudent to have systematic processes whereby data quality is reviewed, monitored, and improved. Additionally, the feasibility of using these systems to support QI should be considered [21, 23, 26, 27]. As healthcare systems are ever evolving, this requires ongoing commitment to assessing quality [8]. Our study enhances the literature by providing a pragmatic demonstration that despite the limitations described above, data from routinely collected databases can be harnessed to reveal gaps in care and opportunities to promote clinical engagement in QI. For example, we found that we could potentially quantify the proportion of patients with each diabetes type and assess anthropometric benchmarks for the patient panels. However, there were significant challenges with data capture for both diabetes type and anthropometric measures which call to question whether this is a clinical practice or EMR documentation issue. Previous studies have demonstrated similar inconsistency in distinguishing types of diabetes in EMRs, suggesting that this may be a system rather than a clinical practice problem [9]. This point can be used as a conversation starter with clinical teams to engage them in QI. The systematic approach used to obtain our results and interrogation of the data has been outlined in detail and could be adopted by other centers looking to do similar work. It also highlights the necessity of engaging a multidisciplinary team if using routinely collected data to inform QI.

This collaborative project by the Physician Learning Program and the Edmonton Zone Diabetes Quality Council is the first stage in supporting data-driven QI for specialist diabetes care in Edmonton. It has illuminated challenges and barriers to using information captured in the shared EMR and administrative databases to improve patient outcomes. The next steps include engaging clinical teams, the EMR front-end users, to prioritize the clinical areas to act on to improve patient care. Current opportunities include assessing whether front-end users are adequately trained and are familiar with the user interface, whether they are incentivized and understand the utility of accurate data capture for a QI program, and developing tools from data, such as dashboards, that could enhance patient care and/or improve efficiency for healthcare workers. It is critical to acknowledge that engaging front-end users alone will not improve data quality and usability; system enablers are crucial to ensure clinicians are not burdened with more data entry tasks which could lead to disengagement and burnout [28]. Administrators and payers must work with clinicians to change daily clinical workflows with the goal of promoting clinically meaningful data capture and use while simplifying the process. This may include capitalizing on opportunities, such as natural language processing and artificial intelligence, which can simplify data capture and integration [26]. It may require the involvement of innovators and researchers from both the academic community and those outside the traditional health information technology realm [26].

## Conclusion

Combining EMR and administrative health data to inform QI initiatives in diabetes specialties clinics at a regional level is possible, albeit challenging. This is a first step to creating an LHS whereby data-driven QI is feasible using data captured in routine clinical care. However, undertaking such work is difficult and requires substantial personnel support as the process of obtaining clinically meaningful data from these sources is not linear, but rather iterative with significant emergent locally contextual limitations. Given these limitations, assumptions that EMR and administrative databases can serve as the foundation of continuous QI must be tempered. Creating clinically actionable data requires clinician champions, data experts, user-friendly EMR design and access, and administrative support. To promote a culture of improvement within an organization and across the healthcare system, QI must be recognized as a continuous operational need that requires dedicated infrastructure and human resources, including clinician time, to ensure its relevance.

## Supplementary Information

**Additional file 1**. Outcomes assessed and the source of data used.

**Additional file 2**. Description of the EMR and administrative databases.

**Additional file 3**. Alberta Health ICD 9 codes used to define and capturecomorbidities [29].

**Additional file 4**. Demographics, process, and clinical outcomes.

## Data Availability

All data generated or analyzed during this study are included in this published article [Additional file 1,2,3,4]. Any additional information can be available from the corresponding author on reasonable request.

## Abbreviations

AHS: Alberta Health Services
ATC: Anatomical Therapeutic Codes
CPSSCN: Canadian Primary Care Sentinel Surveillance Network
DISER: Diabetes Infrastructure for Surveillance, Evaluation, and Research
EMR: Electronic medical record
GDM: Gestational Diabetes Mellitus
HHS: Hyperglycemic hyperosmolar state
ICD: International Classification of disease
LHS: Learning health system
nonHDL-C: non high density lipoprotein cholesterol
NAPCReN: Northern Alberta Primary Care Research Network
PHN: Personal health number
PIN: Pharmaceutical Information Network
PLP: Physician Learning Program
QI: Quality improvement

## Declarations

Taylor McGuckin: Nothing to disclose.

Dr. Campbell-Scherer reports personal fees from Pfizer, grants from NOVAD (Univ. Hospital Foundation, Úlberta Gov. & Novo Nordisk), CIHR, Alberta Innovates - Health Solutions, Alberta Government (Physician Learning Program)Ð outside the submitted work .

Brock Setchell: Nothing to disclose.

Dr. Peter Senior: Dr. Senior reports payments for clinical services from the Alberta Academic Medicine and Health Services Plan. He was section chief for endocrinology in the Edmonton Zone and co-chair of the Edmonton Zone Diabetes Quality Council when the work was conducted.

Dr. Roseanne O Yeung: Dr. Yeung reports personal fees from Merck, personal fees from Diabetes Canada, personal fees from Novo Nordisk, personal fees from Sanofi, grants from Astra Zeneca, grants from Allergen, outside the submitted work.
