# Supplementary material for: Real word challenges in integrating electronic medical record and administrative health data for regional quality improvement in diabetes: a retrospective cross-sectional analysis
Source: BMC Health Serv Res. 2023 Jan 2;23:1. doi: 10.1186/s12913-022-08882-7 (PMC9806899; doi:10.1186/s12913-022-08882-7)
Supplement: Supplementary file 1 — Additional file 1. Outcomes assessed and the source of data used. [file 12913_2022_8882_MOESM1_ESM.docx]

Appendix 1: Outcomes assessed and the source of data used

| **Clinical Category** | **Variables and Definitions** | **Data source** |
| --- | --- | --- |
| **Demographics** | Sex | EMR |
|  | Age |  |
| **Service use** | Number of outpatient visits and unique individuals | EMR |
|  | New versus follow-up visits |  |
|  | Type of visit: in person, phone, email/letter, other (Abstract, BPA, Episode Changes, History, Lab Results, Orders Only, Referral, and Refill) |  |
|  | Length of scheduled appointments |  |
| **Diabetes type** | Type 1 Diabetes Mellitus | EMR |
|  | Type 2 Diabetes Mellitus |  |
|  | Gestational Diabetes Mellitus |  |
|  | Uncertain: Includes all diagnostic combinations where we could not be confident in determining an individual’s diagnosis (e.g., coded as having both Type 1 Diabetes Mellitus and Type 2 Diabetes Mellitus) |  |
| **Anthropomorphic data/biometrics*** | Body mass index: Calculated from the last height recorded from any visit in 2014 to 2018 and most recent weight from 2017 or 2018 visit. Includes the proportion of individuals whose BMI could be calculated. | EMR |
|  | Blood pressures: Mean blood pressure of individuals at in-person visits. Includes the proportion of visits with a blood pressure result and proportion at target (<130/80 mmHg). |  |
| **Clinical markers and laboratory results*** | Hemoglobin A1c: Proportion of individuals with hemoglobin A1c <7.0%. | AHS Labs |
|  | Albumin: creatinine ratio: Used to assess albuminuria, the number of individuals with at least one test result, and the number of individuals dispensed an ACE inhibitor or angiotensin receptor blocker (ARB) based on albumin: creatinine ratio results.  Includes proportion of individuals with albumin: creatinine ratio (ACR) <2mg/mmol |  |
|  | Estimated glomerular filtration rate: Used to assess individuals’ most recent results and proportion with at least one test result.  Includes proportion of individuals with estimated glomerular filtration rate (eGFR) ≥ 60 mL/min |  |
|  | LDL cholesterol: Used to assess albuminuria, the number of individuals with at least one test result, and the number of individuals dispensed a statin based on LDL results.  Includes proportion of individuals with LDL <2.0 mmol/l |  |
|  | Non-HDL cholesterol: Used to assess individuals’ most recent results and proportion with at least one test result.  Includes proportion of individuals with non HDL <2.6 mmol/l |  |
|  | Triglycerides: Used to assess individuals’ most recent results and proportion with at least one test result.  Includes proportion of individuals with triglycerides TG < 1.5 mmol/L |  |
| **Comorbidities and complications** | Diabetic ketoacidosis: Visit to any medical center 1 year prior to any of their diabetes clinic visit dates with diagnosis of Alberta Health ICD 9 Codes 250.1, 250.10, 250.11, 250.19 | AHS physician claims |
|  | Hyperglycemic hyperosmolar state (HHS): Alberta Health does not capture this code in claims data |  |
|  | Hypoglycemia: Visit to any medical center 1 year prior to any of their diabetes clinic visit dates with diagnosis of Alberta Health ICD 9 Codes 251.0 and 251.2 |  |
|  | Diabetes related kidney disease: Visit to any medical center 1 year prior to any of their diabetes clinic visit dates with diagnosis of Alberta Health ICD 9 Codes 250.3, 250.30, 250.31, 250.39 and/or eGFR<60mL/min/1.73m^2^ and/or albumin creatinine ratio ≥2mg/mmol |  |
|  | Cardiovascular disease: Lifetime history of cardiovascular disease Alberta Health ICD codes 410-413, 414.0, 414.0, 414.8, 414.9, 428*, 429.2, 440.0, 440.9 |  |
|  | Perivascular disease: Lifetime history of perivascular disease Alberta Health ICD codes 440.2, 443.8, 443.9, 444.2, 250.6, 250.60, 250.61, 250.69 |  |
|  | Ischemic stroke: Lifetime history of ischemic stroke Alberta Health ICD codes 431, 433, 433.1, 433.2, 433.3, 433.8, 433.9, 434, 434.1, 434.9, 435 |  |

*Targets per 2018 Diabetes Canada guidelines [27]
