# Supplementary material for: Real word challenges in integrating electronic medical record and administrative health data for regional quality improvement in diabetes: a retrospective cross-sectional analysis
Source: BMC Health Serv Res. 2023 Jan 2;23:1. doi: 10.1186/s12913-022-08882-7 (PMC9806899; doi:10.1186/s12913-022-08882-7)
Supplement: Supplementary file 2 — Additional file 2. Description of the EMR and administrative databases. [file 12913_2022_8882_MOESM2_ESM.docx]

Appendix 2: Description of the EMR and administrative databases

| **Database** | **Description** |
| --- | --- |
| eClinician | An EMR platform that was used at all the diabetes outpatients clinics in the Edmonton zone. It is an “integrated information management platform supporting the collection, access, use, and sharing of information supporting the delivery of health services to persons and populations in multiple settings across the continuum of care” [28]. It captures information that can be used to inform patient care. |
| Physician Claims | Captures “claims submitted for payment of Alberta service providers for health services delivered under the Alberta Health Care Insurance Plan.” It includes patient, provider, and service information such as health service code, date of service, amount paid, facility, up to three diagnostic codes, and shadow billed claims (service data is optionally submitted by physicians on alternative payment plans). Coded using the Alberta Health international Classification of Disease (ICD) codes (Appendix 3). |
| AHS Labs | Houses lab results from the 3 Lab Information Systems used in Alberta. Results are stored in both standardized and unstandardized (e.g., free text) formats from inpatient, outpatient, and community settings. |
