# Supplementary material for: Real word challenges in integrating electronic medical record and administrative health data for regional quality improvement in diabetes: a retrospective cross-sectional analysis
Source: BMC Health Serv Res. 2023 Jan 2;23:1. doi: 10.1186/s12913-022-08882-7 (PMC9806899; doi:10.1186/s12913-022-08882-7)
Supplement: Supplementary file 3 — Additional file 3. Alberta Health ICD 9 codes used to define and capturecomorbidities [29]. [file 12913_2022_8882_MOESM3_ESM.docx]

Appendix 3: Alberta Health ICD 9 codes used to define and capture comorbidities [29]

| **Condition** | **ICD Codes** | **Code Name** |
| --- | --- | --- |
| Diabetic ketoacidosis | 250.1 | Diabetes with ketoacidosis |
|  | 250.10 | Diabetes with ketoacidosis - Adult-onset type |
|  | 250.11 | Diabetes with ketoacidosis - Juvenile Type |
|  | 250.19 | Diabetes with ketoacidosis - Unspecified whether adult-onset or juvenile type |
| Hyperosmolar hyperglycemic state | Not in captured by Alberta Health |  |
| Hypoglycemia | 251.2 | Other disorders of pancreatic internal secretion - Hypoglycemia, unspecified |
|  | 251.0 | Other disorders of pancreatic internal secretion - Hypoglycemia coma |
| Diabetes-related chronic kidney disease | 250.3 | Diabetes with renal manifestations |
|  | 250.30 | Diabetes with renal manifestations - Adult-onset type |
|  | 250.31 | Diabetes with renal manifestations - Juvenile type |
|  | 250.39 | Diabetes with renal manifestations - Unspecified whether adult-onset or juvenile type |
| Cardiovascular diseases | 410 | Acute myocardial infarction |
|  | 411 | Other acute and subacute forms of ischaemic heart disease |
|  | 412 | Old myocardial infarction |
|  | 413 | Angina pectoris |
|  | 414.0 | Other forms of chronic ischaemic heart disease - Coronary atherosclerosis |
|  | 414.8 | Other forms of chronic ischaemic heart disease - Other specified forms of chronic ischemic heart disease |
|  | 414.9 | Other forms of chronic ischaemic heart disease - Unspecified |
|  | 428* | Heart failure |
|  | 440.0 | Atherosclerosis - Of aorta |
|  | 440.9 | Atherosclerosis - Generalized and unspecified |
| Peripheral vascular disease | 440.2 | Atherosclerosis - Of arteries of the extremities |
|  | 443.8 | Other peripheral vascular disease - Other specified peripheral vascular diseases |
|  | 443.9 | Other peripheral vascular disease - Unspecified |
|  | 444.2 | Arterial embolism and thrombosis - Of arteries of the extremities |
|  | 250.6 | Diabetes mellitus - Diabetes with peripheral circulatory disorders |
|  | 250.60 | Diabetes mellitus - Diabetes with peripheral circulatory disorders - Adult-onset type |
|  | 250.61 | Diabetes mellitus - Diabetes with peripheral circulatory disorders - Juvenile type |
|  | 250.69 | Diabetes mellitus - Diabetes with peripheral circulatory disorders - Unspecified whether adult-onset or juvenile type |
| Ischemic stroke | 431 | Intracerebral haemorrhage |
|  | 433 | Unspecified intracranial haemorrhage |
|  | 433.1 | Unspecified intracranial haemorrhage - Carotid artery |
|  | 433.2 | Unspecified intracranial haemorrhage - Vertebral artery |
|  | 433.3 | Unspecified intracranial haemorrhage - Multiple and bilateral |
|  | 433.8 | Unspecified intracranial haemorrhage - Other specified precerebral artery |
|  | 433.9 | Unspecified intracranial haemorrhage - Unspecified |
|  | 434 | Occlusion of cerebral arteries |
|  | 434.1 | Occlusion of cerebral arteries - Cerebral embolism |
|  | 434.9 | Occlusion of cerebral arteries - Unspecified |
|  | 435 | Transient cerebral ischaemia |
