# Supplementary material for: Real word challenges in integrating electronic medical record and administrative health data for regional quality improvement in diabetes: a retrospective cross-sectional analysis
Source: BMC Health Serv Res. 2023 Jan 2;23:1. doi: 10.1186/s12913-022-08882-7 (PMC9806899; doi:10.1186/s12913-022-08882-7)
Supplement: Supplementary file 4 — Additional file 4. Demographics, process, and clinical outcomes. [file 12913_2022_8882_MOESM4_ESM.docx]

Appendix 4: Demographics, process, and clinical outcomes

|  | **Type of Diabetes** | | | |
| --- | --- | --- | --- | --- |
|  | **Type 1** | **Type 2** | **GDM** | **Uncertain^1^** |
| Unique individuals n=11714  n (% of unique individuals) | 2244 (19.2) | 3232 (27.6) | 3542 (30.2) | 2696 (23.0) |
| Clinic visits n=77782  n (% of visits) | 18841 (24.2) | 19140 (24.6) | 27983 (36.0) | 11863 (15.3) |
| Females n=7966, 68%  n (% of unique individuals) | 1187 (14.9) | 1508 (18.9) | 3542 (44.5) | 1729 (21.7) |
| Males n=3748, 32%  n (% of unique individuals) | 1057 (28.2) | 1724 (46.0) | n/a | 967 (25.8)* |
| Mean age (SD) | 37.7 (15.5) | 57.0 (13.8) | 32.5 (4.8) | 45.8 (17.3) |
| In person visits  n (% of visits) | 7471 (39.7) | 10249 (53.5) | 14747 (52.7) | 5515 (46.5) |
| Phone visits  n (% of visits) | 7289 (38.7) | 3729 (19.5) | 3608 (12.9) | 3321 (28.0) |
| Mean appointment length^ females, minutes (SD) | 59.2 (75.2) | 37.3 (45.0) | 46.3 (63.7) | 38.5 (50.4) |
| Mean appointment length^ males, minutes (SD) | 72.5 (88.2) | 43.9 (50.5) | n/a | 61.9 (65.3) |
| Height and weight recorded  n (% unique individuals) | 1129 (50.3) | 1940 (60.0) | 3168 (89.4) | 691 (25.6) |
| Mean BMI (SD) | 27.4 (5.6) | 33.7 (7.7) | 32.5 (6.5) | 31.0 (7.5) |
| BP measured  n (% of in person visits) | 2637 (35.3) | 5477 (53.4) | 11599 (78.7) | 2581 (46.8) |
| <130/80mmHg n (%) | 1421 (53.9) | 3265 (59.6) | 9305 (80.2) | 1757 (68.1) |
| HbA1c measured  n (% unique individuals) | 2164 (96.4) | 3186 (98.6) | 2452 (69.2) | 2193 (81.5) |
| <7.0% n (%) | 319 (14.7) | 665 (20.9) | 2427 (99.0) | 942 (43.0) |
| ACR measured  n (% unique individuals) | 2130 (94.9) | 3029 (93.7) | 593 (16.7) | 1655 (61.3) |
| <2mg/mmol n (%) | 1698 (79.7) | 1582 (52.2) | 494 (89.3) | 961 (58.1) |
| eGFR measured  n (% unique individuals) | 1707 (76.1) | 2518 (77.9) | 2646 (74.7) | 1898 (70.4) |
| ≥60 mL/min n (%) | 1556 (91.2) | 1875 (74.5) | 2642 (99.8) | 1599 (84.2) |
| HDL females  (% unique individuals) | 1187 (52.9) | 1508 (46.7) | n/a^2^ | 1729 (64.1) |
| HDL males  (% unique individuals) | 1057 (47.1) | 1724 (53.3) | n/a^2^ | 967 (35.9) |
| non-HDL  (% unique individuals) | 1315 (58.6) | 1961 (60.7) | n/a^2^ | 1065 (39.5) |
| <2.6mmol/L n (%) | 181 (13.8) | 328 (16.7) | n/a^2^ | 151 (14.2) |
| LDL  (% unique individuals) | 1872 (83.4) | 2649 (82.0) | n/a^2^ | 1599 (59.3) |
| <2mmol/L n (%) | 670 (35.8) | 1413 (53.3) | n/a^2^ | 586 (36.6) |
| Triglycerides  (% unique individuals) | 2066 (92.1) | 3131 (96.9) | n/a^2^ | 2214 (82.1) |
| <1.5 mmol/L n (%) | 1641 (79.4) | 1227 (39.2) | n/a^2^ | 1129 (51.0) |
| Diabetic ketoacidosis (DKA)  n (% unique individuals) | 283 (12.6) | 149 (4.6) | n/a^2^ | 234 (8.7) |
| Hypoglycemia  n (% unique individuals) | 121 (5.4) | 80 (2.5) | 3 (0.1) | 101 (3.7) |
| Diabetes kidney disease  n (% unique individuals) | 432 (19.3) | 1451 (44.9) | 96 (2.7) | 696 (25.8) |
| Cardiovascular disease  n (% unique individuals) | 336 (15.0) | 1349 (41.7) | 120 (3.4) | 686 (25.4) |
| Peripheral vascular disease  n (% unique individuals) | 101 (4.5) | 249 (7.7) | 2 (0.1) | 166 (6.2) |
| Ischemic stroke  n (% unique individuals) | 67 (3.0) | 311 (9.6) | 10 (0.3) | 179 (6.6) |
| ^1^Used when an individual had more than one diabetes type recorded that could not be differentiated (e.g, type 1 and type 2 diabetes mellitus)  *At 39 and 48 visits, males were coded as having Diabetes in Pregnancy and/or Gestational Diabetes, respectively. These individuals have been grouped into the Uncertain diabetes type category  ^Appointment lengths were available for clinic visits, appointments, consult letters, and chronic disease management. As such, some visit types, such as classes, will inflate the mean and median appointment lengths  ^2^These measures are not clinically indicated or relevant for individuals with GDM  GDM - Gestational diabetes mellitus, BMI - body mass index, BP - blood pressure, HbA1c - hemoglobin A1c, ACR - albumin: creatinine ratio, eGFR - estimated glomerular filtration rate, LDL - low density lipoprotein cholesterol, HDL - high density lipoprotein cholesterol, non HDL - non high density lipoprotein cholesterol | | | | |
